# Detection and Characterization of CD133^+^ Cancer Stem Cells in Human Solid Tumours

**DOI:** 10.1371/journal.pone.0003469

**Published:** 2008-10-21

**Authors:** Virginia Tirino, Vincenzo Desiderio, Riccardo d'Aquino, Francesco De Francesco, Giuseppe Pirozzi, Umberto Galderisi, Carlo Cavaliere, Alfredo De Rosa, Gianpaolo Papaccio

**Affiliations:** 1 Dipartimento di Medicina Sperimentale, Sezione di Istologia ed Embriologia, Seconda Università degli Studi di Napoli, Napoli, Italy; 2 Dipartimento di Oncologia Sperimentale, I.N.T. Pascale, Napoli, Italy; 3 Dipartimento di Medicina Sperimentale, Sezione di Biotecnologie, Seconda Università degli Studi di Napoli, Napoli, Italy; 4 Dipartimento di Medicina Pubblica e Preventiva, Sezione Anatomia Umana, Seconda Università degli Studi di Napoli, Napoli, Italy; 5 Dipartimento di Scienze Odontostomatologiche Ortodontiche e Chirurgiche, Secondo Ateneo di Napoli, Napoli, Italy; Baylor College of Medicine, United States of America

## Abstract

**Background:**

Osteosarcoma is the most common primary tumour of bone. Solid tumours are made of heterogeneous cell populations, which display different goals and roles in tumour economy. A rather small cell subset can hold or acquire stem potentials, gaining aggressiveness and increasing expectancy of recurrence. The CD133 antigen is a pentaspan membrane glycoprotein, which has been proposed as a cancer stem cell marker, since it has been previously demonstrated to be capable of identifying a cancer initiating subpopulation in brain, colon, melanoma and other solid tumours. Therefore, our aim was to observe the possible presence of cells expressing the CD133 antigen within solid tumour cell lines of osteosarcoma and, then, understand their biological characteristics and performances.

**Methodology and Principal Findings:**

In this study, using SAOS2, MG63 and U2OS, three human sarcoma cell lines isolated from young Caucasian subjects, we were able to identify and characterize, among them, CD133^+^ cells showing the following features: high proliferation rate, cell cycle detection in a G2\M phase, positivity for Ki-67, and expression of ABCG2 transporters. In addition, at the FACS, we were able to observe the CD133^+^ cell fraction showing side population profile and forming sphere-clusters in serum-free medium with a high clonogenic efficiency.

**Conclusions:**

Taken together, our findings lead to the thought that we can assume that we have identified, for the first time, CD133^+^ cells within osteosarcoma cell lines, showing many features of cancer stem cells. This can be of rather interest in order to design new therapies against the bone cancer.

## Introduction

Osteosarcoma is the most common primary tumour of bone. It occurs in bone and extra osseous sites, and displays a bimodal age distribution, with a first peak during the second decade of life, related to the adolescent growth spurt (400 new paediatric cases per year in the Unites States) and a second peak in older adults [1]. The incidence is slightly higher in African-Americans than in Caucasians and death is usually the result of progressive pulmonary metastasis with respiratory failure due to widespread disease [2]. Sarcoma genetic alterations include both oncosuppressor and oncogene pathways, whose products regulate cell cycle progression [3]. Actually, it is well known that solid tumours are populated by heterogeneous cell populations that include cells with stem-like properties, such as high proliferation rate, quick expansion and invasive growth [4], [5].

A tumour can be envisaged as a whole organ, formed by different cells displaying distinct roles in the economy of the tumour. It is well known that the function of stem cells is to maintain and repair tissues. Stem potential can be also acquired by cancerous cell and this event is very important for tumour progression.

Current opinion is that tumours may derive from a small number of cells having stem-like characteristics. New therapies targeting these cells, which are fundamental for tumor progression, could significantly improve clinical treatment of cancer. Therefore, it is of paramount importance to identify, within tumours, subpopulations of cells exhibiting significant differences in terms of proliferation, stem marker expression and behaviour.

The CD133 antigen is a pentaspan membrane glycoprotein, characterized by two independent studies [6], [7] and originally identified in neuroepithelial stem cells [8]. Its interest as a cancer stem marker has grown dramatically since it appeared that it was able to identify a cancer initiating subpopulation in brain [9] and colon [10]. Moreover, CD133^+^ cells have also been found in hepatocarcinoma [11] and melanoma [12] but, up to now, not yet in osteosarcomas, in which the presence of supposed stem-like cells forming spheres has been reported [13].

Therefore, we aimed to use the CD133 as a marker to detect the possible presence of cancer stem cells within SAOS2, MG63 and U2OS human sarcoma cell lines isolated from osteosarcomas of young Caucasian subjects. These cell lines have been previously used as models of osteosarcoma [14], [15] and osteoblast-like cells [16], [17]. In this study, in order to specifically identify CD133^+^ cancer stem cells, we have investigated the relationship between stem features and the kinetics of the CD133 marker expression.

Our results, first of all demonstrate, for the first time, that the CD133 antigen is observable in cells in different osteosarcoma stabilized cell lines. Moreover, we have shown that these cells display high proliferation rate and that they are capable of forming cluster spheres. In addition, we have found that these cells are highly clonogenic and tumorigenic. Taken together, our data lead to the thought that cancer stem cells (CSCs), that are tumour initiating cells, can be found in osteosarcomas and this may be of paramount importance when designing new anti-cancer therapies for bone.

## Results

**Figure 1:**
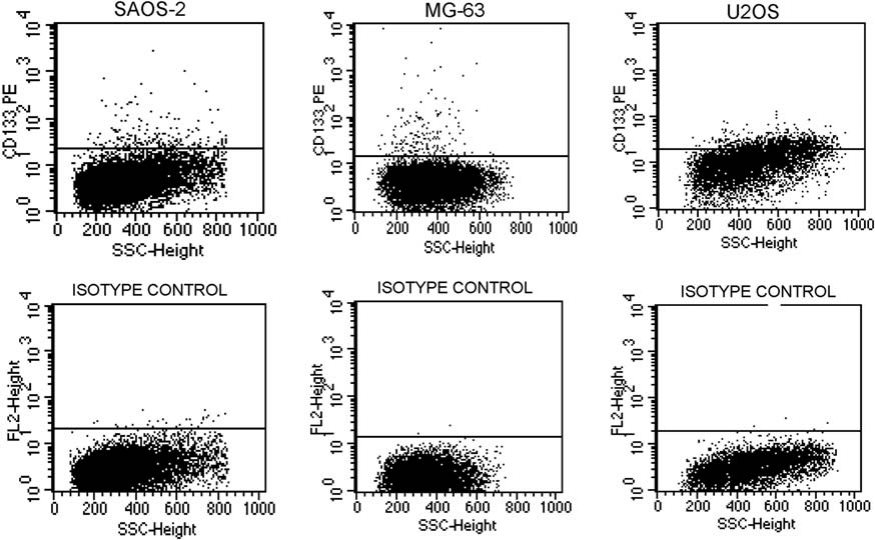
Cytometric analyses for CD133 on SAOS2, MG63 and U2OS cell lines. A CD133^+^ cell population can be detected in all the three cell lines.

SAOS2, U2OS and MG-63 osteosarcoma cell lines were tested in order to detect, within them, the presence of a CD133^+^ cell population. CD133 is a stem cell marker described for the first time in neuro-endothelial progenitors, and recently has been supposed to be a selective marker for Cancer Stem Cells (CSC) in some cancer types. Our results clearly show that in all of the three cells lines, two subpopulations: a CD133^+^ (ranging from 3% to 5%) and a CD133^−^, can be identified (Fig. 1). Using the FACsorting, we obtained a CD133^+^ enriched population (98.8%) and a CD133^−^ cell population. The expression of the CD133 antigen was analyzed by using two different antibodies. Results confirmed that the CD133 negative cells did not express the antigen on the cell surface and that the CD133 positive cells were expressed at the same percentage level using both antibodies.

**Figure 2:**
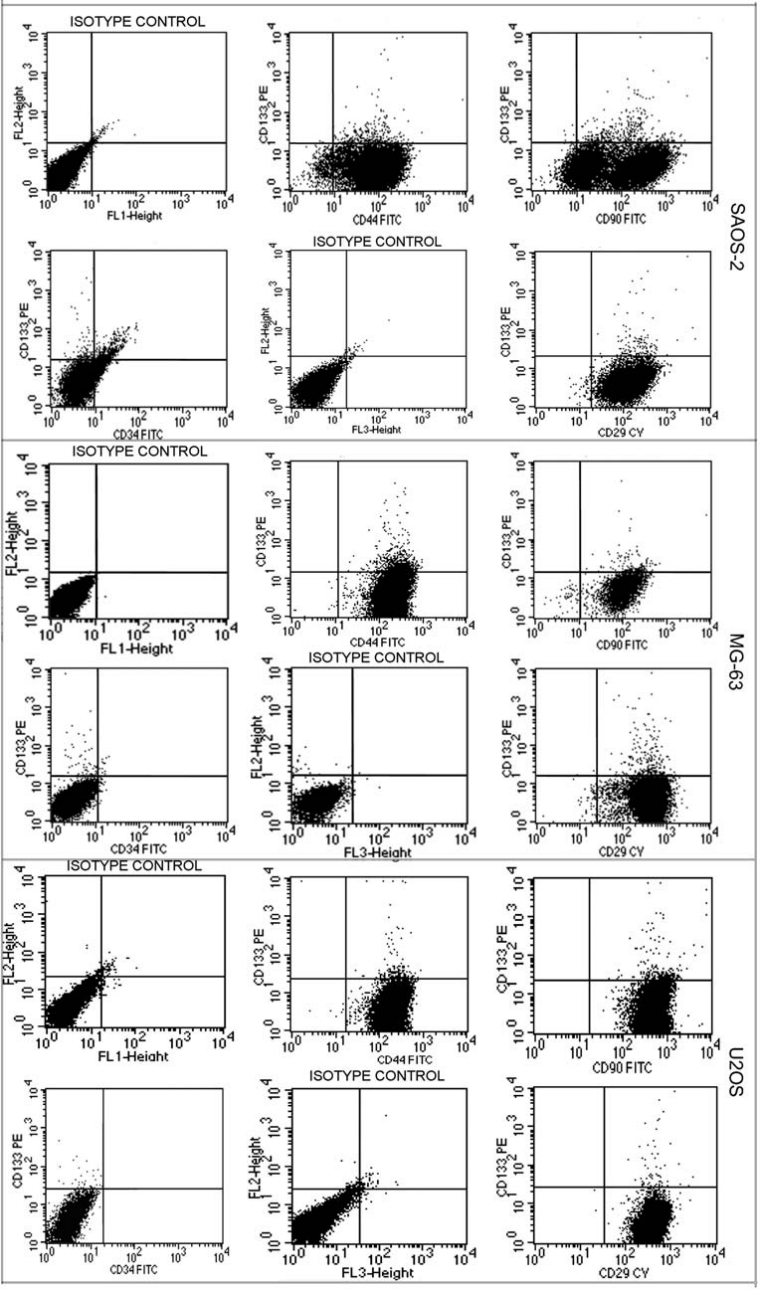
Cytometric analyses for CD133, CD44, CD90, CD34 and CD29 on SAOS2, MG63 and U2OS cell lines. All three cell lines are negative for CD34 antigen but they evidence equal strong positivity for CD29, CD44 and CD90.

Besides, all the three cell lines were negative for CD34 antigen but they evidenced equal strong positivity for CD29, CD44 and CD90, mesenchymal and cancer stem cells markers. Both the fractions of CD133^+^ and CD133^−^ cells were positive for CD29, CD44 and CD90 antigens in all three cell lines (Fig. 2).

The two cell populations (CD133^+^ and CD133^−^) were then used to perform cell cycle analysis, growth analysis, sphere cluster formation assay, soft agar assay and side population detection.

### Cell cycle, Proliferation assays and Growth analyses

**Figure 3:**
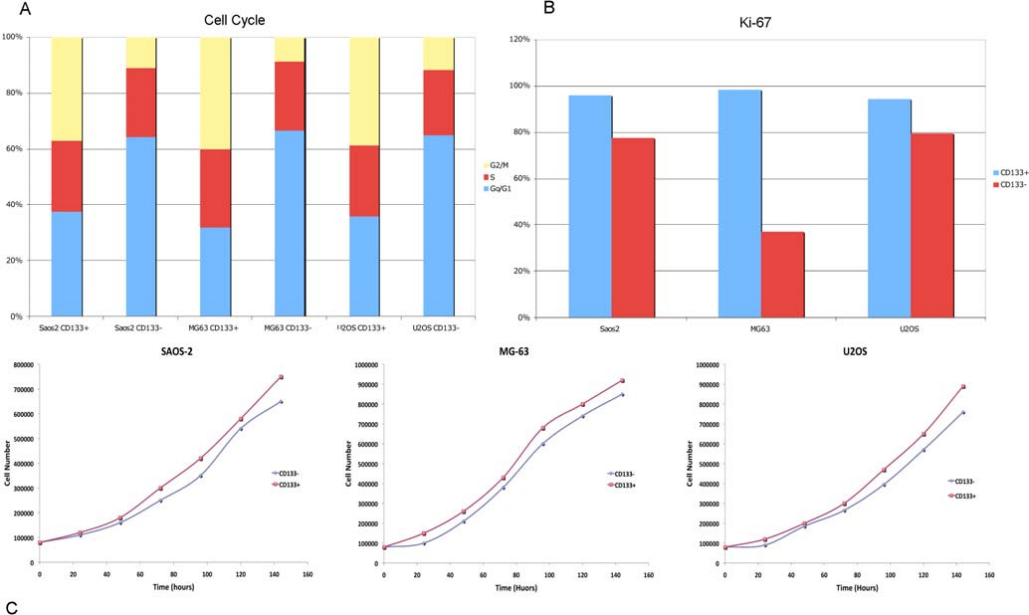
Cell cycle, proliferation and growth analyses. (A) Figure of cell cycle analyses performed on SAOS2, MG63 and U2OS cell lines. A CD133^+^ cell population is mainly observable in the G2\M phase, whereas CD133^−^ cells were predominantly in G0\G1; (B) Figure showing Ki-67 reactivity. CD133^+^ cells resulted to be Ki-67^+^, whereas CD133^−^ were mainly negative for this marker; (C) Figure showing growth curves of CD133^+^ cells with respect CD133^−^ cells in SAOS2, MG63 and U2OS cell lines. CD133^+^ cells possess a high proliferative potential in all the three cell lines.

Propidium Iodide (PI) assay showed a marked difference in the cell cycle of CD133 sorted cells. CD133^+^ cells were mostly in the G2\M phase, while CD133^−^ cells were predominantly in G0\G1 (Fig. 3A), indicating that the CD133^+^ subpopulation is the active proliferating cell fraction. Moreover, all the CD133^+^ cells resulted to be Ki-67^+^ (Fig. 3B) while the majority of CD133^−^ cells were negative for this marker. Ki-67 is a protein expressed only in proliferating cells, thus our results confirm that the CD133^+^ fraction is the source of newly generated cells.

In addition, in order to show whether CD133^+^ cells were capable of extensivelly proliferate when compared to CD133^−^ cells, we observed their growth. Our data showed that the tumour cultures derived from CD133^+^ cells display higher proliferative potential with respect to CD133^−^ cells in all the three cell lines. In fact (Fig 3C) CD133^+^ cells exhibited a mean doubling time of approximately 40 h, 33 h and 38 h in SAOS2, MG63 and U2OS cell lines respectively, whereas CD133^−^ cells showed a mean doubling time of 48 h, 44 h and 42 h in SAOS2, MG63 and U2OS cell lines respectively. When cells were adherent, after sorting, the percentage of CD133 expressing cells significantly decreased (p<0.001) with time of culture (data not shown).

### Sphere Cluster Formation

**Figure 4:**
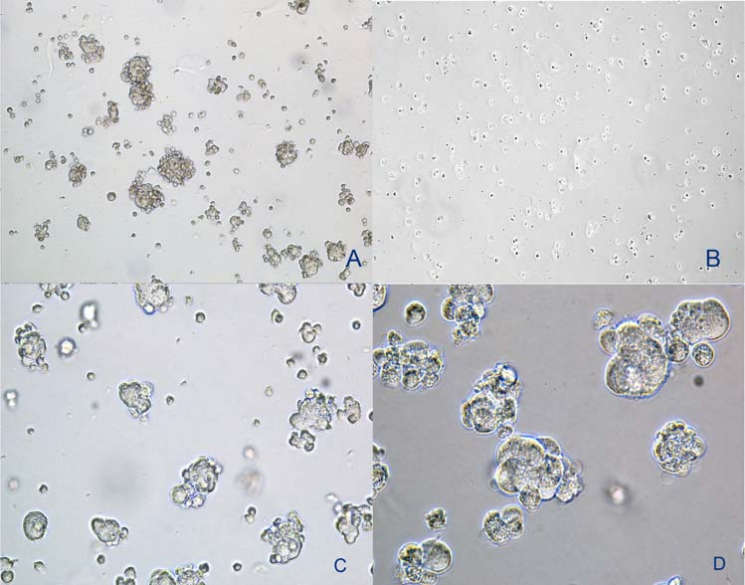
Spheres assay. (A) Sphere clusters formed by CD133^+^ cells in semisolid medium after 24 hours (Original Magnification ×100); (B) CD133^−^ cells in semisolid medium after 7 days, do not form spheres. (Original Magnification ×100); (C) Sphere clusters formed by CD133^+^ cells after 48 hours (Original Magnification ×200); (D) Sphere clusters formed by CD133^+^ cells after a new sorting (Original Magnification ×400).

The ability to grow in suspension in serum free medium, described for the first time to select neural stem cell through neurosphere formation, has been largely investigated as a tumor initiating cell selection method. Glioblastoma, colon cancer, and melanoma cells above all, selected for their ability to form sphere clusters, were found to be highly tumorigenic and able to propagate and reconstitute original tumor architecture when injected into permissive hosts. Our results on osteosarcoma cell lines indicated that sphere clusters were clearly observed already after 24 h in CD133^+^ cultures (Fig. 4A,C,D), while CD133^−^ did not form spheres (Fig. 4B). After 7 days of culture, spheres obtained in CD133^+^ cells were seeded in standard plates with 10% FBS. Cells migrated from the spheres within a few hours and adhered to the bottom of the flasks, assuming a polygonal shape. These cells resulted to be smaller in size, compared with CD133^−^ cells. After a week, we performed an additional test for the CD133 antigen on adherent cells. Interestingly, again a CD133^−^ population was observed, providing evidence that CD133^−^ cells, at this time, derive from CD133^+^ cell. Then, we performed a new sorting of these cells and observed that the CD133^+^ fraction still retained the ability to form spheres, while the CD133^−^ did not.

**Figure 5:**
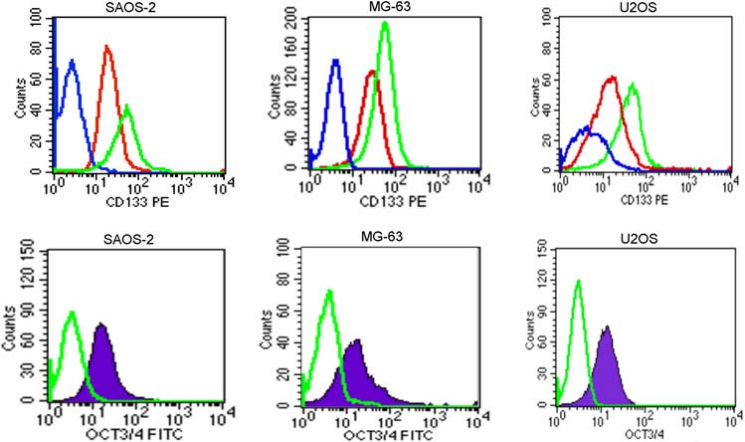
Cytometric analyses for CD133 and OCT3/4 on sarcospheres in SAOS2, MG63 and U2OS. The blue line indicates isotype controls, red and green lines indicate the expression of CD133 at the 4^th^ and 6^th^ cell passage, respectively. In the histograms, OCT3/4 expression is analyzed at the 6^th^ cell passage; the green line indicates isotype controls. Sarcospheres both in CD133 and OCT3/4 are strongly positive.

In addition we tested OCT3/4 and CD133 expressions at 6^th^ cell passage and at 4^th^ and 6^th^ passage, respectively on sarcospheres derived from CD133^+^ cells after sorting. Results showed that the spheres were enriched both in OCT3/4 and CD133 with time of culture (Fig. 5).

### Soft Agar Assay

Soft agar assays were performed in order to observe differences in cell tumorigenicity by means of the ability of CD133^+^ cells to form colonies with respect CD133^−^ cells. As shown in Table 1, colonies were formed more efficiently by CD133^+^ cells, which gave rise to a 5.5±1.8-fold larger number of colonies than those detected in CD133^−^ population (*p*<0.005) in SAOS2; 7.7±1.5-fold larger number of colonies than those observed in CD133^−^ population (*p*<0.001) in MG63, and 6.8±1.7-fold larger number of colonies than those observed in CD133^−^ population in U2OS (P<0.005).

**Table 1:** Colony-forming efficiency of CD133^+^ cells versus CD133− population^a^ in SAOS2, MG63 and U2OS cell lines.

		N° colonies	% of formed colonies^b^	Fold increase^c^
SAOS2	CD133+	5,5±1,8	5,7±1,9	4,2±0,9
	CD133−	1,3±0,4	1,4±0,4	1
MG63	CD133+	7,7±1,5	8,1±1,4	4,8±0,7
	CD133−	1,6±0,5	1,7±0,6	1
U2OS	CD133+	6,8±1,7	7,3±1,8	4,5±0,9
	CD133−	1,5±0,4	1,7±0,5	1

a Results are the mean±standard devition of three experiments from different cases.

b Represents the number of colonies with respect to the number of wells plated in experiments.

c Ratio between the percentage of colonies formed by CD133+ versus CD133− cells.

### Side population and ABCG2

**Figure 6:**
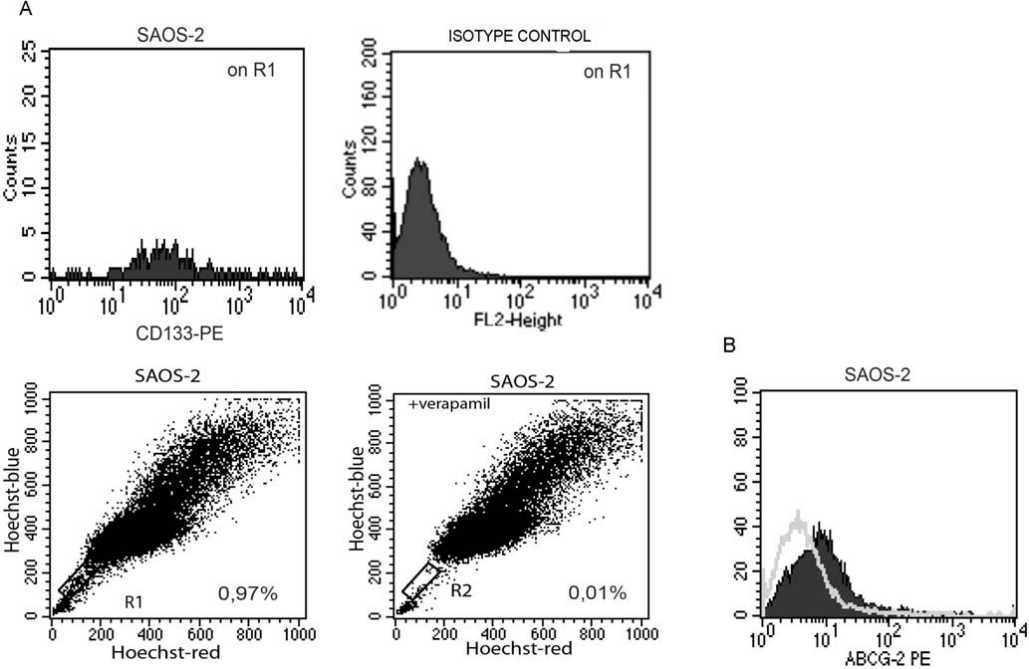
Side population analysis. (A) Cytometric analyses of the side population. The CD133^+^ fraction includes a small subset (0.97%), expressing the characteristic profile of a side population at FACS. (B) ABCG2 expression in SAOS2 cell line, showing an evident positivity; the grey line indicates the isotype control.

Within the CD133^+^ fraction a very small subset (0.97%) expressed the characteristic profile of a side population. It is known that the side population phenotype is the most significant attribute of cancer stem cells. In this study we show for the first time that in osteosarcoma cell lines a side population can be detected. Moreover, we found that all the three cell lines expressed the ABCG2 transporter (Fig. 6) which are usually associated with side population phenotype and drug resistance.

### Immunohistochemistry and immunofluorescence

**Figure 7:**
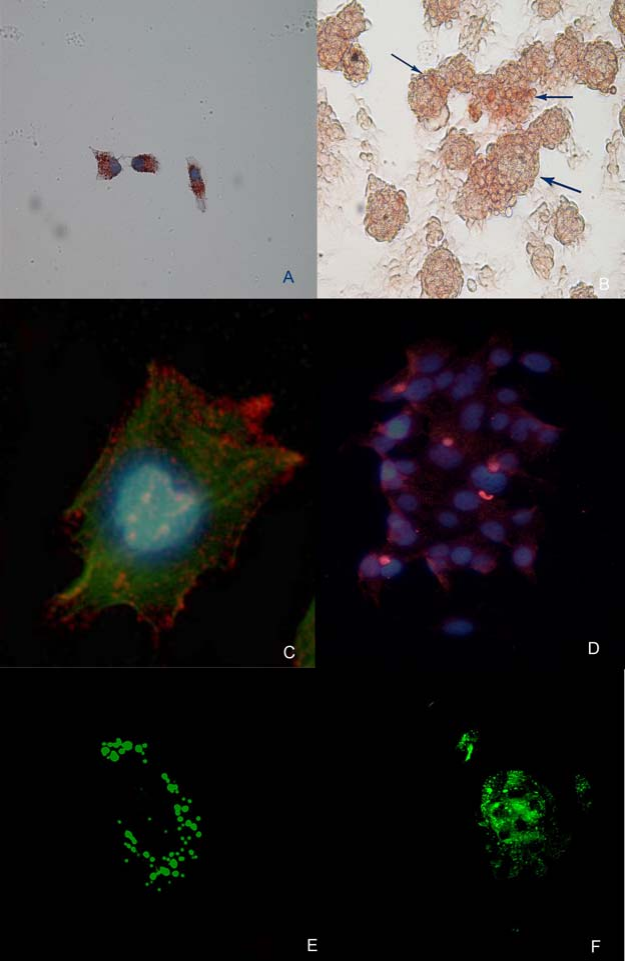
CD133 expression in adherent cells and floating spheres. (A) Immunohistochemical analyses on adherent cells and (B) floating spheres showing the presence of CD133 antigen (arrows). (Original Magnification. ×100); (C) Immunofluorescence analysis on SAOS-2 for CD133 PE, cytoskeleton is stained with phalloidin-FITC, nucleus with DAPI (Original Magnification. ×400); (D) Immunoflurescence analysis on SAOS-2 spheres for CD133 PE after 24 hours in adhesion. (Original Magnification ×200); (E) Confocal analyses on adherent cells and (F) floating spheres confirming the presence of the CD133 antigen. (Original Magnification. ×400).

**Figure 8:**
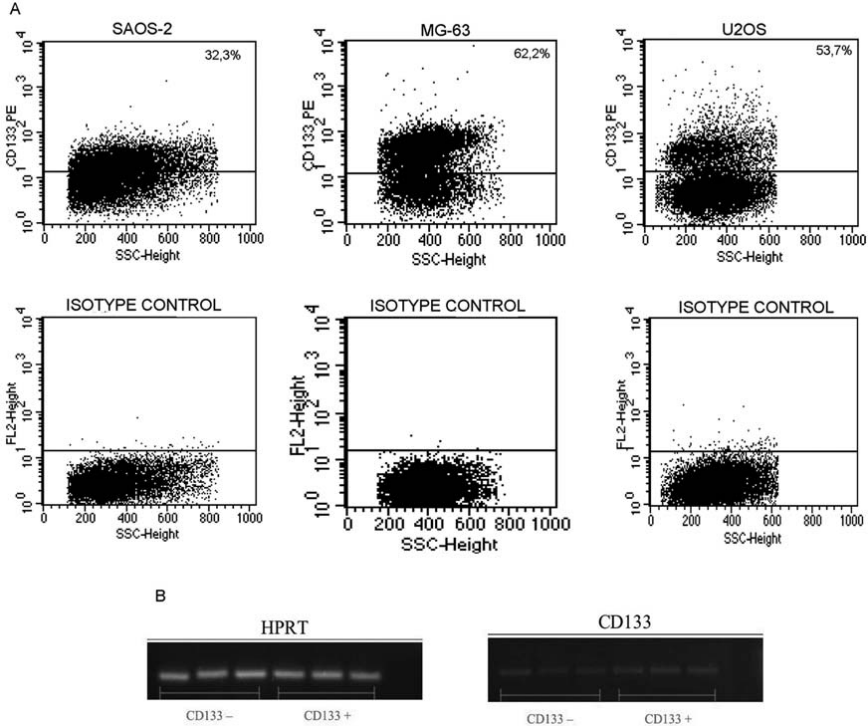
CD133 intracellular expression in adherent cells. (A) Figure performed at FACS showing intracellular expression of CD133 antigen in SAOS-2, MG-63 and U2OS. (B) Figure showing at the Real time PCR the mRNA transcript expression in CD133^+^ and CD133^−^ cells. The levels are almost identical as detected by in both CD133 positive and negative cells.

Both immunohistochemical and immunofluorescence analyses on adhered cells and floating spheres were performed in this study. Our results, accordingly with FACS analyses, confirmed the presence of the CD133 antigen on the cell membrane of CD133^+^ sorted cells. Moreover, floating spheres showed a widely diffuse staining for CD133, corroborating the fact that spheres are formed by CD133^+^ cells. Interestingly, the CD133^−^ sorted fraction were stained for CD133, that was localized within intra-cytoplasmic vesicles as shown at confoal microscopy (Fig. 7A,B,C,D,E,F). In order to deeply understand this aspect, we performed, at the FACS, an intra-cellular staining for CD133. We found that in all of the three cell lines an intracellular positivity was observed (Fig. 8A), while control isotypes were negative.

### Real Time-PCR analysis

The expression of CD133 mRNA levels in CD133^+^ and CD133^−^ adherent cells were almost identical as detected by Real Time PCR (Fig. 8B). This confirms both FACS and immunofluorescence results, as shown above.

## Discussion

Osteosarcoma is a highly aggressive tumour, which prevailingly affects young people. Following recent studies [10]–[13], [18] supporting the presence of a highly tumorigenic cell subset – commonly called Cancer Stem Cells (CSCs) – within the tumour bulk, we tried to isolate and characterize this subpopulation in osteosarcoma cell lines. Tumour lesions encompass heterogeneous cell populations, among which the presence of stem antigens can be evidenced by means of phenotypical analyses. The presence of a CD133^+^ subpopulation within human solid tumours has been documented by several reports [10]–[13], [18]–[21], and cells expressing this marker seem to be different from the other cancerous cells. In particular, CD133^+^ cells possess stem-like features, such as differentiation ability, high proliferation rate, sphere cluster formation and the ability to propagate tumour in permissive hosts. Cancer therapy failures may be due to inefficient effects of current therapy upon potentially quiescent CSCs, which remain vital and retain the capacity to regenerate the tumour [22], [23]. Development of new CSC-targeted strategies is currently hindered by the lack of reliable markers for the identification of CSCs and the poor understanding of their behaviour and fate. Cell lines, derived from tumours, retain hierarchical stem cell patterns, demonstrable with different clonogenic abilities, related to cellular properties, such as size, adhesiveness, dye exclusion and gene expression patterns [24].

In this study, we used the CD133 antigen as a cancer stem-cell marker in order to identify, within stabilized osteosarcoma cell lines, cells exhibiting different properties in terms of proliferation rate, clonogenic efficiency, sphere-cluster formation and dye exclusion. Screening three osteosarcoma cell lines, CD133^+^ subsets were found to be 3% to 5% of total cells, according with the assumption that CSCs should be only a very small cell subset. There are no differences in the CD29, CD44 and CD90 expressions both in CD133^+^ and CD133^−^ cells. Actually, cancer stem cells are considered to be quiescent *in vivo* and they rarely divide asymmetrically, giving birth to highly proliferating progeny [5]. Nevertheless, if a stem hierarchy does exist in cell lines, cells with stem features cannot be quiescent; otherwise, they should be lost in a few passages. According with this consideration, we found that CD133^+^ cells were highly proliferative, when compared with CD133^−^ cells, as confirmed by PI analysis, Ki-67 labelling and growth analyses.

CD133^+^ cells showed many differences with respect to their negative counterparts, having the ability to grow as spheres in a semisolid medium and to efficiently form colonies in soft agar. These assays are commonly regarded respectively as indexes of self-renewal, tumorigenicity and clonogenicity. Moreover, sarcospheres, which derived from CD133^+^cells, expressed high levels of OCT3/4, that are transcription factors critically involved in self-renewal and in the maintenance of pluripotency of undifferentiated embryonic stem cells [25]. In our hands the CD133^+^ cells showed all those abilities and expressed ABCG2, which is a membrane transporter, usually associated with side population phenotype and drugs resistance [26]. Therefore, this can be considered to be an additional marker for CSCs. In addition, in this study, we effectively showed, for the first time, that a side population can be detected also in osteosarcoma cell lines. The side population is defined by Hoechst exclusion in flow cytometry [27], [28]. It represents only a small fraction of the whole cell population and expresses high levels of various members of the ABC transporter family , such as ABCG2 and MDR1, which are responsible for drug resistance [29], [30]. As expected, we found that the side population was made by a very small fraction (0.97%) of the total cells, and, interestingly, it was completely included within the CD133^+^ subset.

Our data, taken together, may lead to the tought that CD133^+^ cells are cancer stem cells. Actually, although this should be supported by an *in vivo* tumour xenograft, as shown in other solid tumours [10]–[13], [18]–[21], unfortunately, we were not able to perform *in vivo* experiments because osteosarcoma cell lines do not graft with any host. We effectively tried to perform *in vivo* transplantation of our CD133 stem cells, but without success, like all the others researchers.

In addition, we may also hypothesize that the CD133^+^ subset encompasses a smaller CD133^+^\ABCG2^+^\SP^+^ population, which could be resistant to drugs, possesses stem features and may effectively drives cancer progression.

Interestingly and unexpectedly, in our hands, some cells that did not express the CD133 marker on the membrane resulted to produce a detectable amount of CD133 mRNA transcripts, and expressed the CD133 antigen in cytoplasmic vesicles as shown by confocal microscopy. Nevertheless, CD133^+^ and CD133^−^ sorted populations clearly display different behaviours. This leads us to speculate on the functional role of CD133 in organizing the membrane and in selecting CSCs, as follows: i) being CD133 highly involved in sphere cluster formation, it is necessary for non-adherent cell growth as well as for cell-to-cell cross talk, which allows cells to communicate and receive stimuli that are normally supplied by adhesion molecules; ii) CD133^+^ and CD133^−^ populations are in continuous and mutual exchange and only true CSCs express CD133 constantly on the membrane.

In conclusion, our findings suggest that CD133 marker may be useful to indicate the differentiated/undifferentiated state of osteosarcoma tumours and this may lead to new approaches in order to design a more specific therapy and ameliorate prognosis.

## Materials and Methods

### Cell culture

SAOS2, MG63 and U2OS were purchased from ATCC CELL BANK; cells were placed in α-MEM culture medium, supplemented with 15% FCS, 100 µM 2P-ascorbic acid, 2 mM L-glutamine, 100 U/ml penicillin, 100 µg/ml streptomycin (all purchased from Invitrogen, San Giuliano Milanese, Milan, Italy) and placed in 75 ml flasks with filtered valves. Flasks were incubated at 37°C in a 5% CO_2_ and the medium changed twice a week. After confluence, cells were subdivided into new flasks until the end of the experiment.

### Flow Cytometry and Cell Sorting

Cells were detached using 0.02% EDTA in phosphate-buffered saline (PBS), counted and washed in 0.1% BSA in PBS. At least 500,000 cells (in 100 µl PBS/0.5% BSA) were incubated with fluorescent-labelled monoclonal antibodies or respective isotype controls (1/10 diluted 4°C for 30 min in the dark). After washing steps, the labelled cells were analyzed by flow cytometry using a FACS Vantage cell sorter (Becton & Dickinson, Mountain View, CA,USA). The antibodies used were mouse anti-human CD133/2 PE conjugated (Miltenyi Biotec S.r.l. Calderara di Reno, Bologna, Italy), mouse anti-human CD133 PE conjugated (eBioscience), mouse anti-human CD29 CY conjugated (BD Pharmingen,Buccinasco,Milan, Italy), mouse anti-human CD34 PE conjugated (Miltenyi Biotec), mouse anti-human CD44 FITC conjugated (Miltenyi Biotec), mouse anti-human CD90 FITC conjugated (BD Pharmingen), mouse anti-human OCT3/4 non conjugated (Santa Cruz Biotechnology, Inc. Santa Cruz, California, U.S.A), mouse anti-human Ki67 FITC conjugated (Miltenyi Biotec) and mouse anti-human ABCG2 non conjugated (Santa Cruz). CD133^+^ cells were sorted for experiments. CD133^−^ cells were harvested as control. The purity of sorted populations was routinely 90%. Isotypes were used as controls.

Seven days after sorting, CD133^−^ cells were detached and tested twice for CD133 expression. OCT3/4 expression was analysed at 6^th^ cell passage (a “passage” indicates that cells are detached when at confluence) whereas CD133 expression was analysed at 4^th^ and 6^th^ cell passage on sarcopheres derived from CD133^+^ cells in the three cell lines.

For intracellular staining of Ki67, CD133 and OCT3/4, cells were processed using the Caltag Fix & Perm Kit (Invitrogen, Milan, Italy) following the manufacturer's guidelines. All data were analyzed using a CellQuest software.

### Hoechst 33342 Exclusion Assay

SAOS2, MG63 and U2OS cells were resuspended at 2.0×10^6^ cells/ml in pre-warmed α-MEM culture medium and divided into two portions. A portion was treated with 50 µM verapamil and the other was left untreated. Both portions were incubated in α-MEM culture medium with 5 µg/ml Hoechst 33342 (Sigma, Milan, Italy) for 90 minutes at 37°C. After incubation the cells were washed in PBS and kept on ice for 5 minutes, and analyzed for Hoechst 33342 efflux by FACS vantage (Becton Dickinson, Milan, Italy) [31]. The Hoechst 33342 dye was excited at 350 nm ultraviolet and resultant fluorescence was measured at two wavelengths using a 424/44 BP and 675 LP filters for detection of Hoechst blue and red, respectively.

### Cell Cycle and Proliferation Analyses

Cell cycle was analysed by flow cytometry. Cells were harvested in PBS containing 2 mM EDTA, washed once with PBS, fixed in iced ethanol 70° and incubated with 50µg/ml PI (Sigma) plus Rnasi 1 mg/ml for 60 min at 4°C in the dark. Stained nuclei were analysed with a FACS Vantage cell sorter (Becton & Dickinson, Mountain View, CA,USA), and the data analysed using a Mod-Fit 2.0 cell cycle analysis programme (Becton-Dickinson).

### Growth analysis

After sorting by CD133, SAOS2, MG-63 and U2OS cells were plated at a density of 8,0×10^4^ cells/well in 6-well plates. Every twelve hours cells were harvested and re-suspended in PBS. An aliquot of cell suspension was diluted with 0.4% trypan blue (Sigma–Aldrich), pipetted onto a haemocytometer and counted under a microscope at 200× magnification. Live cells excluded the dye, whereas dead cells admitted the dye and consequently stained intensely with trypan blue. The number of viable cells for each experimental condition was counted and represented on a linear graph. The doubling time (DT) was determined from the growth curves or by using the formula:
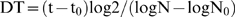
where t and t_0_ were the times at which the cells were counted, and N and N_0_ were the cell numbers at times t and t_0_, respectively [32].

### Soft Agar Assay

In order to assay the different tumorigenic potential into two cell fractions, CD133^+^ and CD133^−^ cells were plated in soft agar at a density of 100, 500 or 1000 cells/well in 24 wells plates in triplicate. Colo 38 melanoma cell line was used as positive control.

For the base layer, 2.4% agar stock solution was melted in a microwave oven, cooled to 40 C in a water bath and then mixed with culture medium to obtain a solution of 0.8% Agar in α-MEM. 0.5 ml/well of this solution was added to 24-well plates. For the top layer, the agar stock solution was diluted with culture medium to obtain a solution of 0.3% agar in α-MEM. 0.5 ml/well of the solution was gently mixed and aliquoted into the 24-well plates. CD133^+^, CD133^−^ and wilde type SAOS-2, MG63 and U2OS cells were successively plated and incubated for 21 days at 37°C in a humidified atmosphere of 5% CO2 in air and 50 µl α-MEM. At the end of the incubation period, colonies were stained with 150 µl/well of NBT (nitrobluetetraziolium) at the concentration of 1 mg/2 ml in PBS, and counted using an inverted microscope (Nikon TS 100, Nikon, Milan, Italy).

### Immunofluorescence Staining

CD133^+^ and CD133^−^ cells cultured in 24-well plates were fixed with a solution of 4% paraformaldehyde/0.2% Triton in PBS for 30 min at 4°C, washed in PBS, treated with PBS/5% milk for 60 min at room temperature and then stained with primary antibodies at 4°C overnight. The primary antibodies used were mouse anti-human CD133/2 PE conjugated (Miltenyi Biotec), mouse anti-human Phalloidin FITC conjugated (AlexaFluor-Invitrogen) incubated for 60 min at 4°C. The nuclei were stained with DAPI. Cells were then washed twice as described above and observed under the fluorescence microscope (Nikon TE 2000-S, Milan, Italy). Isotypes and non-probed cells were used as controls.

### Sphere Assays

Cells were plated at a density of 60,000 cells/well in 6-well ultra low attachment plates (Corning Inc., Corning, NY, USA) in DMEM/F12 cell medium, supplemented with 1% methylcellulose, progesterone (10 nM), putrescine (50 µM), sodium selenite (15 nM), transferrin (13 µg/ml), insulin (10 µg/ml; Sigma) and human EGF (10 ng/ml) and human bFGF (10 ng/ml; Sigma) [13]. Fresh aliquots of EGF and bFGF were added every other day. After culture for 48–72 hours, spheres were visible at inverted phase-contrast microscope (Nikon TS 100, Nikon).

### Laser-scanning confocal microscopy

Cells grown in 24 well plates were fixed with 4% paraformaldehyde for 30 min at 4°C, washed in PBS, treated with PBS/5% milk for 60 min at room temperature and then incubated with primary antibodies at 4°C over night. The primary antibody was a mouse anti-human CD133/1 Pure (Miltenyi Biotec); the secondary antibody (goat anti-mouse FITC or PE conjugated AbCAM) was incubated for 60 min at 4°C, and the DAPI, used to stain the nucleus, was incubated for 7 minutes at room temperature. The same procedure was performed on sarcospheres. All labelled cells were stored at 4°C before images acquisition, using a Zeiss Laser-scanning confocal microscope LSM 510 Meta (Zeiss- Oberkocken-Germany). Images were captured at a resolution of 512×512 pixels. The appropriate argon laser fluorescence for visualization of the CD133 was used with an excitation wavelength of 488 nm and emission filter BP 505–530.

### Immunohistochemistry

Immunoistochemistry for CD133 on SAOS2, MG63 and U2OS cells was performed. Cells were plated at a density of 50,000 cells/well in 24 well plates, fixed with 3.5% paraformaldehyde for 10 min at 4°C, and washed in PBS. Primary antibody used was mouse anti-human CD133/1 Pure (Miltenyi Biotec). For secondary antibody and staining, the DAKO Cytomation En Vision+System-HRP kit (AEC) was used according to the manufacturer's instructions. The nuclei were stained with haematoxylin and the cells were observed under an inverted light microscope. This procedure was also performed on sarcospheres.

### RT-Real time PCR

Sequences for mRNAs from the nucleotide data bank (National Center for Biotechnology Information, USA) were used to design primer pairs for RT-PCR reactions (Primer Express, Applied Biosystems, CA, USA). Primer sequences are available on request. Appropriate regions of HPRT (Hypoxanthine-guanine phosphoribosyltransferase) cDNA were used as controls. PCR cycles were adjusted to have linear amplification for all the targets. Each RT-PCR reaction was repeated at least 3 times. The Real Time PCR assays were run on Opticon 4 machine (MJ, Research, Waltham, MT, USA). Reactions were performed according to the manufacturer's instructions by using SYBR green PCR Master mix. Primer sequences were designed with Primer express software.

All the above described experiments were performed in quadruplicates

### Statistical analysis

Student *t*-test was used for statistical evaluation. Level of significance was set at p<0.05.
