# Correction: Detection and Characterization of CD133^+^ Cancer Stem Cells in Human Solid Tumours

**DOI:** 10.1371/annotation/6475ab87-5c24-4ee6-9989-0762186dd073

**Published:** 2008-12-23

**Authors:** Virginia Tirino, Vincenzo Desiderio, Riccardo d'Aquino, Francesco De Francesco, Giuseppe Pirozzi, Antonio Graziano, Umberto Galderisi, Carlo Cavaliere, Alfredo De Rosa, Gianpaolo Papaccio, Antonio Giordano

Drs. Antonio Graziano and Antonio Giordano were not included in the author byline. Antonio Graziano should be listed as the sixth author and is affiliated with Dipartimento di Scienze Odontostomatologiche, Ortodontiche e Chirurgiche, Secondo Ateneo di Napoli, Napoli, Italy. His contributions were as follows: Performed the experiments.

Antonio Giordano should be listed as the 11th author and is affiliated with Sbarro Institute for Cancer Research and Molecular Medicine, Center of Biotechnology, College of Science and Technology, Temple University, Philadelphia, Pennsylvania, United States of America, and Department of Human Pathology and Oncology, University of Siena, Siena, Italy. His contributions were as follows: Conceived and designed the experiments, contributed reagents/materials/analysis tools. 

